# Chemical Characterization of Kraft Lignin Prepared from Mixed Hardwoods

**DOI:** 10.3390/molecules26164861

**Published:** 2021-08-11

**Authors:** Ji-Sun Mun, Justin Alfred Pe, Sung-Phil Mun

**Affiliations:** 1 Department of Carbon Materials and Fiber Engineering, Jeonbuk National University, Jeonju 54896, Korea; jismun20@jbnu.ac.kr; 2 Department of Wood Science and Technology, Jeonbuk National University, Jeonju 54896, Korea; justinpe@jbnu.ac.kr

**Keywords:** kraft lignin, acacia, mixed hardwood, milled wood lignin, structural analysis

## Abstract

Chemical characterization of kraft lignin (KL) from mixed hardwoods (*Acacia* spp. from Vietnam and mixed hardwoods (mainly *Quercus* spp.) from Korea) was conducted for its future applications. To compare the structural changes that occurred in KL, two milled wood lignins (MWLs) were prepared from the same hardwood samples used in the production of KL. Elemental analysis showed that the MWL from acacia (MWL-aca) and mixed hardwood (MWL-mhw) had almost similar carbon content, methoxyl content, and C_9_ formula. KL had high carbon content but low oxygen and methoxyl contents compared to MWLs. The C_9_ formula of KL was determined to be C_9_H_7.29_O_2.26_N_0.07_S_0.12_(OCH_3_)_1.24_. The M_w_ of KL and MWLs was about 3000 Da and 12,000–13,000 Da, respectively. The structural features of KL and MWLs were investigated by Fourier transform infrared spectroscopy (FT-IR) and nuclear magnetic resonance spectrometry (^1^H, ^13^C NMR). The analyses indicated that KL underwent severe structural modifications, such as γ-carbon cleavage, demethylation, and polycondensation reactions during kraft pulping, which resulted in increased aromatic content and decreased aliphatic content. The main linkages in lignin, β-O-4 moieties, were hardly detected in the analysis as these linkages were extensively cleaved by nucleophilic attack of SH^-^ and OH^-^ during pulping.

## 1. Introduction

Lignin is the second-most abundant organic carbon source after cellulose. The amount of lignin discharged is about 50–70 million tons annually [1,2]; however, it has been estimated that only a small amount (ca. 1–2%) is used in specialty products [3]. Lignin can be obtained by pulping and kraft pulping accounts for about 85% of the total lignin production [4]. The lignin recovered from the black liquor after kraft pulping is called kraft lignin. The majority of KLs are directly burned in pulp mills to generate energy and to recover the remaining pulping chemicals, while some KLs are used as rubber additives and phenolic resin adhesives [5,6]. Limitations on the utilization of KLs are due to their heterogeneity, modified structure, presence of sulfur from sodium sulfide, and the poor quality of the final product [7,8].

Along with KL, lignosulfonates (LS) are commercially available lignins that have been used for a long time in the production of vanillin, dispersant, coagulant, etc. [9]. Although KL is less utilized than LS, KL is still an attractive resource for researchers and pulping companies since it is cheap, renewable, and available in large amounts. Therefore, converting KL into a high value-added material is one major research goal, even though the use of KL is currently limited. Studies on the application of KL as bioadhesives [10], supercapacitors [11], fuel for fuel cells [12], polyurethane foams [13], and carbon fibers [14] have been conducted recently. However, few applications were commercially profitable [8], and further studies are needed for the mass consumption of KL. In addition, converting lignin into value-added products is one way to unleash its potential [7]. Particularly, in the near future, massive amounts of lignin will be derived as a by-product since many researchers are trying to use lignocellulosic biomass for the production of bioethanol or biosugar. If a beneficial way to use KL is developed, the utilization of less structurally modified lignins from bioethanol and biosugar production will become easier as well.

Since KL is an underutilized resource, the purpose of this study was to investigate the chemical characteristics of KL from mixed hardwoods to proceed to further applications involving new value-added lignin-based products, such as carbon materials, fine chemicals, and dyestuffs. Most studies on the characterization of KL focused on softwood KL rather than hardwood KL. Hence, this study reports the characterization of KL from mixed hardwoods. Moreover, little emphasis was given to the comparison of hardwood KL and MWLs with respect to the structural changes that occurred in lignin. Therefore, in this study, KL was compared to MWLs prepared from the same hardwood samples used for kraft pulping.

## 2. Results and Discussion

### 2.1. Chemical Composition of Acacia and Mixed Hardwood

The results of alcohol-benzene extract and total lignin contents of acacia and mixed hardwood are shown in Table 1. The alcohol-benzene extract contents were 1.51% for acacia and 1.60% for mixed hardwood, showing slightly comparable values. The total lignin content of acacia was 26.90% which was slightly higher than mixed hardwood (24.76%). In general, it is known that the lignin content of tropical hardwoods is comparable to that of temperate softwoods [9]. As acacia species are grown in tropical or subtropical regions like Vietnam, the total lignin content of acacia was higher.

### 2.2. Elemental Composition of KL and MWLs

The C, H, O, N, S, and OCH_3_ content of KL and MWLs are summarized in Table 2. The methoxyl content was estimated using the integration ratio of protons derived from the aromatic and the methoxyl groups in the ^1^H NMR as described by Abreu [15]. KL was not only chemically modified during kraft pulping, but new elements were introduced. Considerable amounts of sulfur were present in KL unlike in the MWLs, as kraft pulping chemical (Na_2_S) was directly involved in lignin depolymerization which generated sulfide ions during pulping. In addition, as shown in Table 2, KL had higher carbon content, and lower oxygen and methoxyl contents compared to MWLs.

The C_9_ molecular formula, C_9_ molecular weight, and double bond equivalent (DBE) of KL and MWLs are listed in Table 3. The DBE approximates the number of double bonds and the presence of cyclic structures together with the frequency of interunit linkages in a phenylpropane (C_9_) unit [16,17]. The calculated DBE values revealed that in comparison to MWLs, KL contained a high degree of unsaturation as a consequence of the transformation of lignin moieties (i.e., β-O-4, β-β, β-1, β-5, 5-5, and 4-O-5) into their condensed forms with newly incorporated double bonds. Table 3 also shows that KL had a lower C_9_ MW than MWLs. These outcomes suggest that the original lignin was depolymerized into lignin fragments which were condensed and contained high degree of unsaturation. These results are also in agreement with well-established reactions of model lignin compounds.

**Figure 1:**
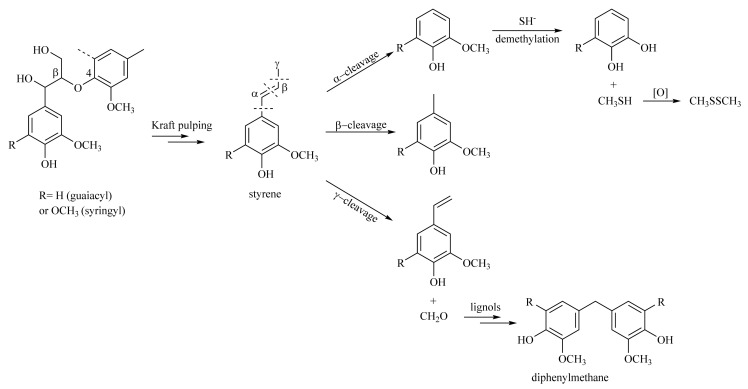
Behavior of β-O-4 linkages during kraft pulping.

Studies on structural modifications during kraft pulping indicated that β-O-4 structures, with hydroxyl or carbonyl groups attached at the α or γ positions, underwent β-ether cleavage, resulting in styrene-like compounds, which then further degraded leading to side chain cleavage at the α, β, or γ-carbon positions as shown in Figure 1 [18,19]. The splitting of the side chain from the C_9_ unit caused a reduced C_9_ MW for KL. Also, linkages involving side chain cleavage at the γ-carbon position produced formaldehyde which eventually reacted with other lignols to form condensed products like diphenylmethane [20]. These condensed products increased the carbon content of KL. The demethylation reaction caused by the attack of sulfide ions to the methoxyl group at C-3 or C-5 resulted in the formation of methylmercaptan (CH_3_SH) and then further oxidation produced dimethyldisulfide (CH_3_SSCH_3_) [21]. The demethylation caused reduced methoxyl content for KL compared to MWLs.

### 2.3. MW Distribution, Average MW, and Polydispersity

**Figure 2:**
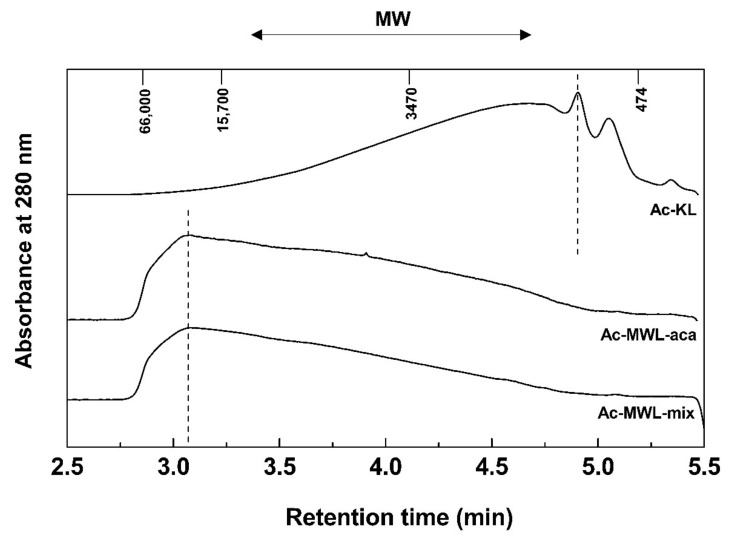
MW distribution of Ac-KL and Ac-MWLs.

The weight-average (M_w_) and number-average (M_n_) MW, and polydispersity index (PDI) of acetylated KL (Ac-KL) and acetylated MWLs (Ac-MWLs) were determined by gel permeation chromatography (GPC). The MW distribution of Ac-KL and Ac-MWLs are shown in Figure 2 while the M_w_, M_n_, and PDI are given in Table 4. As shown in Figure 2, the MW distributions of KL and MWL were very different. In the case of KL, the low MW (less than 10,000) fractions were predominant, and in particular, two peaks were observed at a MW of 1000 or less. On the other hand, the MW distribution of the two types of MWLs had no remarkable difference.

As shown in Table 4, the M_w_ of KL and MWLs was about 3000 Da and 12,000–13,000 Da, respectively. These MWs were similar to those of the previously reported hardwood KL and MWL [22]. KL had a M_w_ of about 1/4 and a M_n_ of 1/3–1/4 with respect to MWLs. Moreover, KL had lower PDI than MWLs. The result indicates that molecules in the original lignin were highly degraded during kraft pulping.

### 2.4. FT-IR Analysis

**Figure 3:**
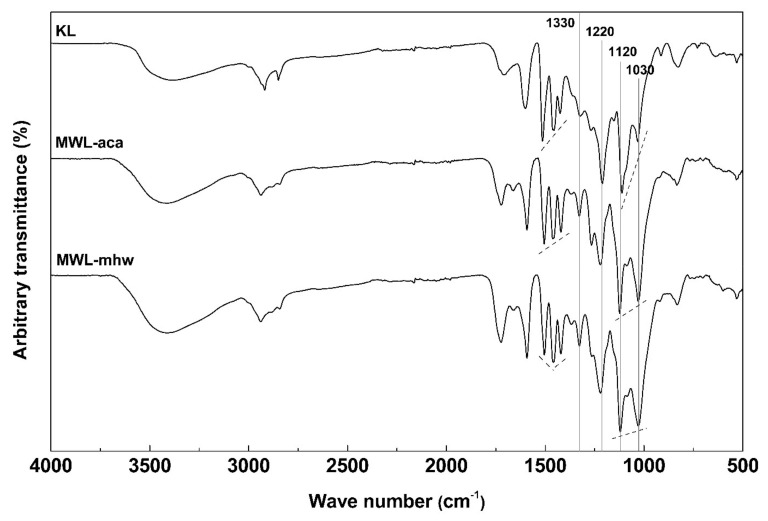
FT-IR ATR spectra of KL and MWLs.

The FT-IR spectra of KL and MWLs are shown in Figure 3 and the assignments, according to Faix [23], are given in Table 5. The overall FT-IR spectral pattern of KL was similar to MWL. However, absorption bands around 1720–1730 cm^−1^, corresponding to ester bonds, were not observed which indicates that the ester bonds were severely cleaved by NaOH and Na_2_S in highly alkaline conditions during kraft pulping. In general, the basic aromatic nucleus structure of hardwood lignin consists of guaiacyl (G) units and syringyl (S) units. In the case of MWL, a distinct absorption band was observed at 1020–1330 cm^−1^, indicating the presence of these units. Specifically, the absorption bands related to the S units appeared around 1325, 1220 and 1120 cm^−1^, and 1275 and 1030 cm^−1^ for the G units. However, for KL, a shoulder appeared at 1275 cm^−1^ and a weak absorption band was observed at 1030 cm^−1^. These results suggest that G and S units were present in KL but might have been significantly degraded or modified compared to MWL. The methoxyl content in KL was low similar to the result of EA. From the FT-IR analysis, it was confirmed that the side chains and aromatic nuclei of KL were chemically modified during kraft pulping.

### 2.5. ^1^H NMR Analysis

**Figure 4:**
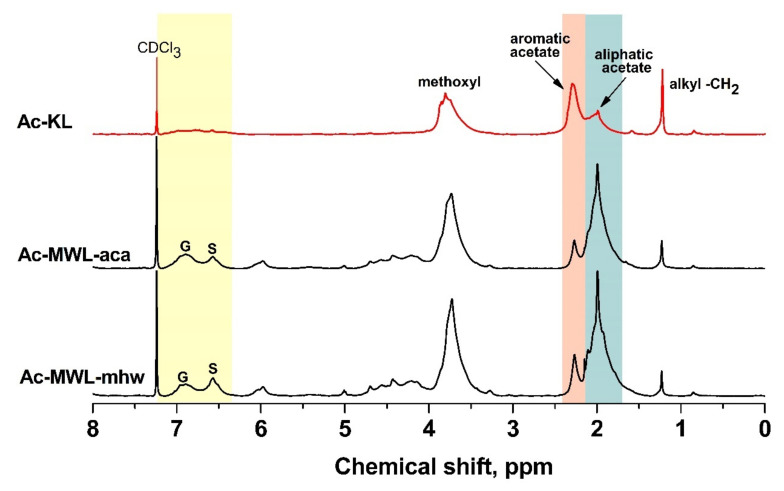
^1^H NMR spectra of Ac-lignins.

The ^1^H NMR spectra of Ac-KL were compared to Ac-MWLs from acacia and mixed hardwood (Figure 4). The aromatic region (7.20–6.25 ppm) revealed the presence of G and S phenylpropane (C_9_) units. Structural changes were shown in the methoxyl (4.00–3.48 ppm), acetyl (2.50–1.60 ppm), and hydrocarbon regions (1.40–0.70 ppm). Table 6 shows the assignments of signal in the ^1^H NMR spectra of Ac-lignins based on literature data [24,25]. The distribution of protons per C_9_ structural unit of Ac-lignins was estimated via integration ratios and their C_9_ molecular formulas [26]. The methoxyl content in the C_9_ molecular formula for KL was 1.24, multiplied by 3 to obtain 3.72, the total number of protons (H) in the methoxyl groups. Integration values of other structural components were made relative to the methoxyl protons in one C_9_ unit. However, some quantitative conclusions could not be confirmed due to overlapping signals, carbohydrate inclusions, and uncertainties in range assignments.

The arylglycerol β-*O*-4 aryl ether linkage (6.25–5.75 ppm, 4.90–4.30 ppm) is the main intermonomeric linkage found in native lignin [24] and is estimated to be about 60–70% of hardwoods. The resonances of the H_α_ and H_β_ in acetylated β-*O*-4 structures at the specified range were hardly detected in Ac-KL as severe β-*O*-4 bond hydrolysis occurred during kraft pulping wherein the β-*O*-4 structures were transformed to styrene-like structures [18]. Further reactions caused bond cleavage at the γ-carbon position, releasing formaldehyde and forming condensation products, such as diphenylmethanes [20].

The aromatic protons per C_9_ unit for Ac-KL were 0.52 for S units and 0.37 for G units, while for Ac-MWL-mhw the aromatic protons per C_9_ unit were 0.84 for S units and 0.57 for G units. Comparing the integration values of Ac-KL to Ac-MWLs, the decreased number of aromatic protons indicated a highly condensed structural motif in KL. The integration values were converted to their molar counterparts to estimate the S to G molar ratio. As shown in Table 7, the S/G molar ratio of Ac-KL was almost similar to Ac-MWL-mhw. A further step of calculation showed the S and G composition in terms of percentage composition. In the case of KL, the % S units were derived from the formula: 3.37/(2.98 + 3.37) × 100, which yielded 53.1%.

The methoxyl protons of Ac-KL (3.72) were 20% less than Ac-MWLs (4.65). The lower amount of methoxyl groups in KL was attributed to demethylation reactions during kraft pulping [21]. The nucleophilic attack of hydrosulfide ions (SH^-^) to aryl methyl ether produced catechol and methylmercaptan. Further oxidation of methylmercaptan produced dimethyldisulfide, which gave KL its characteristic odor.

The number of aliphatic and phenolic hydroxyl groups per C_9_ unit was determined from the corresponding acetyl group signals (2.50–1.60 ppm). For KL, the OAc/OCH_3_ mole ratio = (2.54 + 2.22)/3.72 = 1.28. Thus, the total OAc/C_9_ ratio = (1.24 OCH_3_) × (1.28 OAc/1 OCH_3_) = 1.59, i.e., the number of aliphatic OAc/CH_3_ = (1.24 OCH_3_/C_9_) × (2.22 OAc/3.72 OCH_3_) = 0.74 and the number of phenolic OAc/CH_3_ = (1.24) × (2.54/3.72) = 0.85. Therefore, the number of aliphatic and phenolic hydroxyl groups per 100 C_9_ units of KL was estimated to be 74 and 85, respectively; 178 and 22 for MWL-aca; 181 and 28 for MWL-mhw. As such, the aliphatic hydroxyl group of KL significantly decreased and the aromatic hydroxyl group significantly increased compared to MWL. It was proposed that new phenolic hydroxyl groups were generated as the methoxyl groups were cleaved during kraft pulping.

The presence of increased hydrocarbons, the majority of which were methylene groups, in Ac-KL (0.76) compared to Ac-MWLs (0.31 and 0.19 for acacia and mixed hardwood, respectively) indicated exposure of the methine (–CH–) or methylene (–CH_2_–) groups after ring opening of β-β pinoresinol moieties and β-5 phenylcoumaran moieties and their condensed derivatives.

The main structural change for KL was evident in the acetyl region, wherein Ac-KL contained more aromatic acetyl protons than Ac-MWLs. Meanwhile, Ac-KL contained significantly less aliphatic acetyl protons compared to Ac-MWLs. The high aromatic and low aliphatic contents of KL could be explained by the kraft pulping reactions, such as the β-O-4 bond hydrolysis and bond cleavage at the β- and γ-carbon position as shown in Figure 1. A decrease in methoxyl content in Ac-KL was due to demethylation reactions during kraft pulping.

### 2.6. ^13^C NMR Analysis

**Figure 5:**
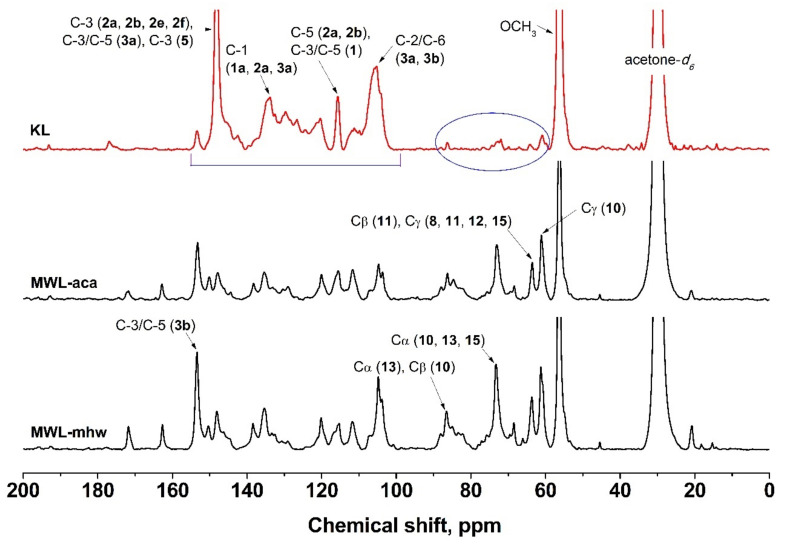
^13^C NMR spectra of KL and MWLs.

The ^13^C NMR spectrum of KL are shown in Figure 5 along with the ^13^C NMR spectra of MWLs from acacia and mixed hardwood which were used as references. The chemical shifts and intensities are listed in Table 8 along with their assignments based on Lüdemann and Nimz [27] and Chen and Robert [28]. Since the ^13^C NMR spectra were recorded under conditions that did not allow quantification, this analysis provided qualitative information only. The ^13^C NMR spectra for MWL-aca and MWL-mhw at the aliphatic and aromatic regions were analogous; however, the ^13^C NMR spectrum for KL showed diminished aliphatic content and elevated aromatic content. This phenomenon was typical for KL since severe structural modifications occurred in the aliphatic and aromatic moieties of lignin during kraft pulping. In addition, demethylation of methoxyl groups and cleavage of γ-carbon leading to polycondensation resulted in distinctive difference between KL and native lignin.

In MWLs, the presence of carboxyl groups from aliphatic esters (primary alcohols) and *p*-hydroxybenzoate were confirmed at 171.5 and 162.5 ppm, respectively, but these peaks were not found in KL since the strongly alkaline environment in kraft pulping easily hydrolyzed the ester bonds.

The aromatic ring resonated in the range of 100 to 160 ppm. The most intense peaks in this region for hardwood MWLs were evident at 103.8–104.9 and 153.4 ppm, which represented the C-2/C-6 and C-3/C-5 of etherified syringyl nuclei. These peaks are specific to hardwood guaiacyl-syringyl (GS) lignins [29]. The peak at 153.4 ppm was weak in KL, suggesting decreased methoxyl content due to demethylation or demethoxylation reactions during kraft pulping. In addition, the C-1/C-4 peak of etherified syringyl nuclei at 138.5 ppm was absent in KL, suggesting a high degree of splitting at the α-carbon position.

On the other hand, the peak at 148.2 ppm was very strong for KL. This peak corresponds to C-3/C-5 of non-etherified S units overlapping with the C-3 of etherified and non-etherified G units. The C-3 of etherified biphenyl (5-5) was also assigned to this peak. Another strong peak from KL at 133.6 ppm was designated to the C-1 of non-etherified *p*-hydroxyphenyl (H), guaiacyl (G), and syringyl (S) nuclei.

**Figure 6:**
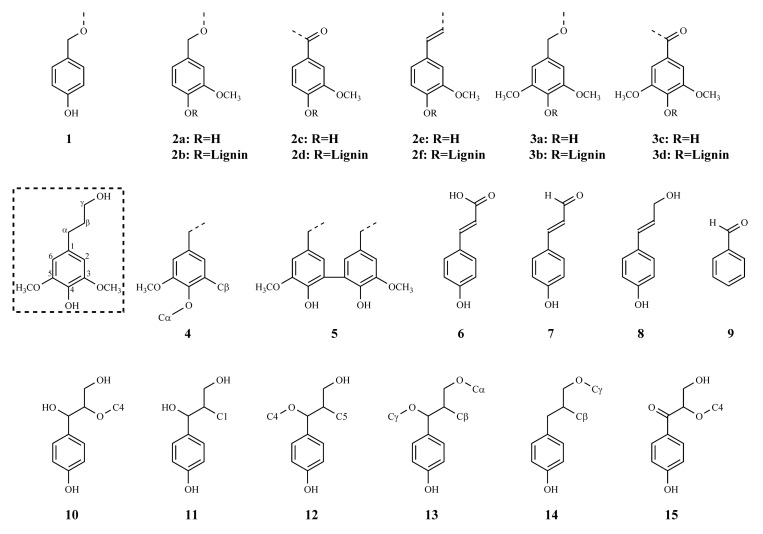
Lignin substructures (**1**–**5**) and side chains (**6**–**15**) corresponding to ^13^C NMR assignments.

The aliphatic groups were situated in the range of 60 to 90 ppm. The moderately strong peaks in this region for hardwood MWLs, other than the β-O-4 structures, were shown at 73.3 and 63.6 ppm. The peak at 73.3 ppm corresponded to the Cα of β-β structures **13**, **14**. The peak at 63.6 ppm indicated Cγ of coniferyl alcohol structures **8**, β-O-1 structures **11**, β-5 structures **12**, and β-O-4 structures with α-carbonyl groups **15**. These peaks were too weak in KL, suggesting loss of these dimeric moieties in KL. The numbered assignments enclosed in a parenthesis in Table 8 denotes the lignin substructures and lignin side chains, which can be found in Figure 6.

In MWLs, the medium to strong peaks at 86.3, 73.3, and 61.2 ppm were assigned to the Cβ, Cα, and Cγ of the β-O-4 structure, respectively. However, these remarkable peaks in MWLs were almost undetectable in KL due to severe cleavage at the α-, β-, and γ-carbons. The cleaved byproducts, such as catechol- or styrene-like structures reacted with the released γ-hydroxymethyl units derived from γ-carbon scission to form highly condensed lignin structures.

## 3. Materials and Methods

### 3.1. Materials

The KL used in this study was kindly provided by Moorim P&P Co., Ltd. (Ulsan, Korea), the only kraft pulp manufacturer in Korea. The wood chips used in kraft pulping were also provided by the same company. Two types of wood chips, namely, from *Acacia* spp. imported from Vietnam and mixed hardwood locally available in Korea (50% *Quercus* spp. + 50% other hardwoods), were ground and wood meals which passed through 40-mesh screen were prepared.

The reagents used were ethanol (HPLC grade), benzene (EP), and acetic anhydride (EP) from Duksan Pure Chemical (Ansan, Korea), 1,2-dichloroethane (GR) from Duksan Pharmaceutical (Ansan, Korea), anhydrous ethyl ether (EP) and acetic acid (EP) from Samchun Chemical (Pyeongtaek, Korea), 1,4-dioxane (HPLC grade) from Wako Chemical (Osaka, Japan), pyridine (GR) from Kanto Chemical (Tokyo, Japan), and toluene (HPLC grade) from Fisher-Scientific Korea (Seoul, Korea). The deuterated solvents used were D_2_O (Merck, Darmstadt, Germany), CDCl_3_ (Eurisotop, Saint-Aubin, France), and acetone-*d*_6_ (Cambridge Isotope Laboratories, Andover, MA, USA). All reagents were used without further purification.

### 3.2. Kraft Pulping and Purification of KL

KL was obtained from kraft pulping spent liquor, which was discharged from Moorim P&P’s chemical pulping mill facility. Pulping conditions and lignin manufacturing processes for KL were provided by Moorim P&P. The mixed wood chips contained 50% acacia and 50% mixed hardwood. Kraft pulping conditions involved 21–23% active alkali (as Na_2_O), 23% sulfidity, and a pulping temperature of 160–165 °C. Lignin was separated and purified from the black liquor obtained under these pulping conditions by acid precipitation method. Sulfuric acid was added to the black liquor so that the final pH of the mixture reached 9.0. Thereafter, after stirring at 70 °C for 1 h, lignin was recovered by centrifugal dehydration. The recovered lignin was washed sequentially with 1.5% dilute sulfuric acid and distilled water (liquid ratio 1:10, 70 °C, 1 h), and was subsequently dried and pulverized into a powdered form. The yield of KL obtained from the black liquor (33% solid content) was 9.7%.

### 3.3. Chemical Composition of Wood Meal

For the determination of the chemical composition and the preparation of MWL, 40 g each of wood meal prepared from acacia and mixed hardwood chips was extracted with 95% alcohol-benzene (1:2) solvent using a large scale Soxhlet apparatus. The wood meal was extracted with a total of 2 L solvent at 70–80 °C for 6 h. After extraction, the solvent was removed in vacuo at 60 °C. The extract was dried overnight in a convection oven at 105 °C. The yield of alcohol-benzene extract was calculated using the equation:

(1) $$\%\;\mathrm{alcohol}-\mathrm{benzene}\;\mathrm{extract}\;=\frac{W_{AB}}{W_{0}}\times 100\;$$

where *W_AB_* = weight of alcohol-benzene extract (g) and *W_0_* = weight of wood meal (g, oven dry weight).

The extractive-free wood meals were air-dried overnight in a fume hood. The wood meals were kept in plastic containers. For MWL preparation, part of the wood meals was thoroughly dried in vacuo in the presence of P_2_O_5_. The lignin content was determined by measuring acid-insoluble lignin (Klason lignin) [30] and acid-soluble lignin [31] in accordance to the TAPPI test method. The total lignin content (TLC) was defined as the sum of acid-insoluble and acid-soluble lignin.

### 3.4. Preparation of MWL

The thoroughly dried extractive-free wood meals were used for preparing MWLs. Briefly, 6 g of wood meal was placed in a 500-mL stainless-steel jar in the presence of toluene. The jar containing the sample was mounted on a vibratory ball mill and treated for 100 h. After milling, the MWLs were prepared according to the Björkman method [32]. The yield of pure MWL was based on the total lignin content and was calculated using the equation:

(2) $$\%\;\mathrm{pure}\;\mathrm{MWL}\;\left(\mathrm{on}\;\mathrm{lignin}\right)=\;\left(\frac{W_{pure}}{TLC\;in\;W_{0}}\right)\;\times 100\;$$

where *W_pure_* = weight of pure MWL (g) and *TLC in W_0_* = total lignin content in wood meal (g). 

The yields of MWL-aca and MWL-mhw, based on total lignin, were 14.17% and 19.22%, respectively.

### 3.5. Acetylation of Lignin

Briefly, 1 g of the thoroughly dried KL and 20 mL of anhydrous pyridine were added into a 100-mL Erlenmeyer flask. The flask was sonicated for 90 s to disperse the mixture. Afterwards, 20 mL of acetic anhydride was added and the mixture was stirred using a magnetic stirrer (SR-306, Advantec, Tokyo, Japan) at room temperature for 48 h. The reaction was quenched by spraying the mixture using a tapered pipette onto an ice bath, containing 350 g of crushed ice and 400 g of deionized water (DI-water) in a 1-L beaker, with vigorous stirring using a magnetic stirrer (RCN-7, Eyela, Tokyo, Japan) for 1 h at room temperature. The Ac-KL was collected by filtration using a nylon 66 membrane filter (47 mm diameter, 0.45 µm pore size, Alltech, Lexington, KY, USA). The Ac-KL on the filter was washed with sufficient DI-water and then dried in vacuo for 3 days in the presence of P_2_O_5_.

For the acetylation of MWLs, 50 mg of MWL was dissolved in 1 mL of pyridine and then 1 mL of acetic anhydride was added dropwise. For MWL precipitation, 17–18 g of crushed ice and 20 g of DI-water were used. The filtering, washing, and drying were carried out in the same manner as for Ac-KL.

### 3.6. Elemental Analysis

C, H, N, and S analysis was performed on moisture-free KL and MWL samples (MWL-aca and MWL-mhw) using an Elemental Analyzer (IT/Flash 2000, Thermo Fisher Scientific, Waltham, MA, USA) at the Center for University-wide Research Facility, Jeonbuk National University (CURF, JBNU). The oxygen composition was calculated as 100 − (C + H + N + S).

### 3.7. Determination of Molecular Weight (MW)

The average MW of lignins was determined by gel permeation chromatography (GPC). A 2 mg of Ac-lignin was dissolved in 1 mL of THF in a 10-mL conical beaker. The beaker was sonicated for 5 s and then filtered through a 0.45 μm PTFE syringe filter (Chemco Scientific, Cheongju, Korea). The filtrate was transferred into a 2-mL vial and diluted 10 times with THF. The GPC (Waters, Milford, MA, USA) was conducted at CURF under conditions shown in Table 9.

### 3.8. FT-IR Analysis

FT-IR analysis was performed on KL and MWL samples using the attenuated total reflection (ATR) method (4000–500 cm^−1^) with an FT-IR spectrophotometer (Frontier, Perkin Elmer, Shelton, CT, USA) at the CURF, JBNU.

### 3.9. ^1^H NMR Analysis

A 15 mg of the Ac-KL sample was dissolved in 0.4 mL of CDCl_3_ in a 10-mL conical beaker. The beaker was sonicated for 1–2 min to dissolve the sample. The mixture was filtered through a fine glass wool suspended inside a Pasteur pipette, which was directly connected to a clean NMR tube. The conical beaker was rinsed with additional 0.3 mL of CDCl_3_ and the contents were transferred as described in previous filtration method. An amount of 10 mg of sample was used for Ac-MWL, and the dissolution and filtration were carried out in the same manner as for the Ac-KL sample. The measurement was conducted using the NMR spectrometer (500 MHz FT-NMR, JNM-ECZ500R, JEOL, Tokyo, Japan) at the CURF, JBNU.

### 3.10. ^13^C NMR Analysis

For KL and MWL-mhw, 100 mg of sample was dissolved in 0.7 mL acetone-*d_6_* and D_2_O (9:1) while for MWL-aca, a different solvent ratio, i.e., 8:2, was used. The filtration was carried out in the same manner as for ^1^H NMR samples. The measurement was conducted using the NMR spectrometer (500 MHz FT-NMR, JNM-ECZ500R, JEOL, Tokyo, Japan) at CURF, JBNU, and a minimum of 10,000 scans were collected.

## 4. Conclusions

KL had higher carbon content but lower oxygen and methoxyl contents than MWLs. The two MWLs, namely, MWL-aca and MWL-mhw, had almost similar elemental composition, methoxyl content, and C_9_ formula. KL had a M_w_ of about 1/4 and a M_n_ of 1/3–1/4 than that of Ac-MWLs. From the results of the elemental analysis, GPC, FT-IR, ^1^H NMR, and ^13^C NMR, it was confirmed that the aromatic nuclei and side chains of KL underwent severe structural modifications, such as γ-carbon cleavage, demethylation, and polycondensation reactions during kraft pulping. Aromatic content increased and aliphatic content decreased in KL. β-O-4 moieties were hardly detected since this linkage was extensively cleaved by nucleophilic attack of SH^−^ and OH^−^ during pulping. Therefore, these results could be the basis for future applications of KL produced in Korea.

## Figures and Tables

**Table 1:** Chemical composition of wood meals used in MWL preparation.

	*Acacia* spp.	Mixed Hardwood
Alcohol-benzene extract (%)	1.51	1.60
Lignin (%)		
Klason	24.69 ± 0.11	21.23 ± 0.25
Acid-soluble	2.21 ± 0.01	3.53 ± 0.09
Total	26.90 ± 0.11	24.76 ± 0.25

**Table 2:** Elemental analyses and methoxyl contents of KL and MWLs.

Lignin Type	Elemental Analyses (%)					
	C	H	O	N	S	OCH_3_
KL	63.12	5.67	28.78	0.48	1.96	19.80
MWL-aca	57.34	5.62	37.04	-	-	21.92
MWL-mhw	57.38	5.75	36.71	0.16	-	21.75

Methoxyl content was calculated by the integrations of the aromatic and methoxyl signals in ^1^H NMR spectrum [15].

**Table 3:** C_9_ formula, C_9_ MW, and DBE of KL and MWLs.

Lignin Type	C_9_ Formula	C_9_ MW (Da)	DBE *
KL	C_9_H_7.29_O_2.26_N_0.07_S_0.12_(OCH_3_)_1.24_	194.9	5.74
MWL-aca	C_9_H_7.61_O_3.56_(OCH_3_)_1.56_	221.2	5.42
MWL-mhw	C_9_H_7.93_O_3.52_N_0.03_(OCH_3_)_1.55_	220.9	5.26

* DBE: double bond equivalent.

**Table 4:** Average MW and PDI of Ac-KL and Ac-MWL.

	M_w_ (Da)	M_n_ (Da)	PDI
Ac-KL	3041	1651	1.8
Ac-MWL-aca	11,898	4416	2.7
Ac-MWL-mhw	13,314	5657	2.4

**Table 5:** Assignment of FT-IR spectra of KL and MWLs.

Peak Range, cm^−1^	Assignment	KL	MWL-aca	MWL-mhw
3412–3460	O–H stretching	○	○	○
2842–3000	C–H stretching in methyl, methylene, and methine groups	2916–2849	2938–2844	2939–2844
1709–1738	C=O stretching in unconjugated ketone and ester group	1731, 1707	1723	1721
1655–1675	C=O stretching in conjugated *p*-substituted aryl ketone	-	1663	1660
1593–1605	Aromatic skeleton vibration plus C=O stretching; S > G: G_condensed_ > G_etherified_	1603	1592	1592
1505–1515	Aromatic skeleton vibration (G > S)	1513	1505	1505
1460–1470	C–H deformations (asymm in –CH_3_ and –CH_2_–)	1457	1462	1457
1422–1430	Aromatic skeleton vibration combined with C-H in plane deformations	1422	1420	1421
1365–1370	Aliphatic C–H stretching in CH_3_ and phenolic OH	-	1369	1367
1325–1330	Condensed S and G ring (G ring bound via position 5)	1320	1328	1327
1266–1270	G ring plus C + O stretching (G-methoxyl C–O)	1267	1266	1265
1221–1230	C–C + C–O + C=O stretching (G_condensed_ > G_etherified_)	1210	1221	1223
1116	Typical for HGS lignins; C=O in ester groups (conj.)	1110	1123	1120
1086	C–O deformation in *sec*-alcohols and aliphatic ether	-	1089	1084
1030–1035	Aromatic C–H in-plane deformation (G > S) + C-O deformation in primary alcohols + C–H stretching (unconjugated)	1034	1029	1029
915–925	C–H out of plane (aromatic ring)	914	-	920
834–835	C–H out of plane in positions (2 and 6 of S units)	-	834	-
817–832	C–H out of plane in positions (2, 5, and 6 of G units)	827	-	832

**Table 6:** ^1^H NMR assignments and distribution of protons per C_9_ structural unit of acetylated lignins.

Range, ppm	Main Assignments	Protons per C_9_ Units		
		Ac-KL	Ac-MWL-aca	Ac-MWL-mhw
7.20–6.80 *	Aromatic proton in G units	0.37	0.84	0.57
6.80–6.25	Aromatic proton in S units	0.52	0.72	0.84
6.25–5.75	H_α_ of β-O-4 and β-1 structures	-	0.31	0.33
4.90–4.30	H_α_ & H_β_ of β-O-4 structures	0.17	1.09	1.09
4.30–4.00	H_α_ of β-β structures, H of xylan residues	0.25	0.85	0.83
4.00–3.48	H of methoxyl groups	3.72	4.68	4.65
2.50–2.22	H of phenolic acetates	2.54	0.65	0.83
2.22–1.60	H of aliphatic acetates	2.22	5.33	5.42
1.40–0.70	Hydrocarbon	0.76	0.31	0.19

* From reference it was 7.25–6.80 but CDCl_3_ solvent peak was detected at 7.24, thus the range was shifted.

**Table 7:** Estimation of S units, G units, and S/G ratio in terms of moles and percentage.

C_9_ Units	In mol			In %		
	Ac-KL	Ac-MWL-aca	Ac-MWL-mhw	Ac-KL	Ac-MWL-aca	Ac-MWL-mhw
G	2.98	6.77	4.59	46.9	59.2	45.5
S	3.37	4.67	5.45	53.1	40.8	54.5
S/G	1.13	0.69	1.19			

**Table 8:** ^13^C NMR assignment of KL and MWLs.

Peak, ppm	Intensity			Assignment
	KL	MWL-aca	MWL-mhw	
195.7	vw	vw	vw	CγHO (**7**), Cα (**15**)
192.6	vw	vw	vw	CαHO (**9**)
171.5	-	w	m	Acetyl C=O in alcohols/phenols
162.5	-	m	m	C-4 in *p*-hydroxybenzoate
153.4	w	s	vs	C-3/C-5 (**3b**)
150.2	-	w	w	C-4 (**2b**), C-3 (**2d**)
148.2	vs	m	m	C-3 (2a,2b,2e,2f), C-3/C-5 (**3a**), C-3 (**5**)
146.3	w	w	w	C-4 (**2a**), Cα (**6**)
144.6	w	w	w	C-4 (**4**)
138.5	-	m	m	C-1/C-4 (**3b**)
135.4	-	m	m	C-1 (**2b**), C4 (**3a**)
133.6	s	w	w	C-1 (**1a**,**2a**,**3a**)
132.5	s	w	w	Cβ (**7**)
129.9	m	vw	vw	C-1 (**2e**,**4**)
129.0	w	vw	vw	C-2/C-6 (**1**), Cβ (**8**)
126.6	m	-	-	C-6 (**2d**)
120.3	m	m	m	C-6 (**2a**,**2b**)
115.5	s	m	m	C-5 (**2a**,**2b**), C-3/C-5 (**1**)
111.9	w	m	m	C-2 (**2a**,**2b**)
109.9	w	-	-	C-6 (**2a**,**2b**)
107.0	w	w	w	C-2/C-6 (**3c,3d**)
104.9–103.8	s	m	s	C-2/C-6 (**3a**,**3b**)
88.1	-	w	w	Cα (**12**)
86.6	-	w	m	Cα (**13**), Cβ (**10**)
86.3	-	w	m	Cβ (**10**)
83.4–81.0	-	w	w	Cβ (**15**)
75.0	w	w	w	Cα (**11**)
73.3	w	s	s	Cα (**10**, **13**, **15**)
63.6	w	s	s	Cβ (**11**), Cγ (**8**,**11**,**12**,**15**)
61.2	-	s	s	Cγ (**10**)
56.4	vs	vs	vs	OCH_3_
20.8	vw	w	m	CH_3_ in acetyl

**Table 9:** Analysis conditions for GPC.

GPC Configuration	Waters (Acquity APC) System, Milford, MA, USA
Columns	Acquity APC 2.5 µm XT 125 (4.6 × 150 mm, Waters, Dublin, Ireland), Acquity APC 1.7 µm XT 45 (4.6 × 150 mm, Waters, Dublin, Ireland)
Flow rate	0.6 mL/min
Sample injection volume	10 μL
Eluent	THF
Column oven temperature	30 °C
Detector	UV (254 nm: polystyrene standards; 280 nm: samples)
Analysis time	10 min
MW polystyrene standards	Blue: 66,000—15,700—3470—474 Da
	White: 35,500—9130—2280—266 Da

## Data Availability

Most of the data used during the preparation of the manuscript are included in the Results and Discussion sections.

## Sample Availability

Samples of the compounds are not available from the authors.
